# Operation in Carcinoma of the Breast

**Published:** 1903-10-17

**Authors:** 


### OPERATION IN CARCINOMA OF THE BREAST.

As time goes by and fresh statistics are collected and published it becomes apparent that there is a slow but progressive advance in the good results obtained by operative measures in carcinoma of the breast. This is very encouraging, for it will doubtless tend more and more towards inducing patients to submit themselves to operation at the earliest possible time. The latest tabulated results are those of Dr. Lewis Stephen Pilcher appearing in the

September number of the *Annals of Surgery*. The cases are arranged according to variations of the technique employed in operating, and the results are arranged correspondingly. There were under his care during the period from 1888 to 1900 inclusive fifty cases in all of primary carcinoma of the breast which were submitted to operation. In seven of these it was found at the operation that the disease had already extended beyond the possibility of entire removal. In the remaining forty-three cases a complete removal of the affected breast and adjacent tissues was performed in accordance with the principle that the incisions should go wide of apparent disease, and that the breast and axillary lymphatics, with the connective tissue and fat in which they are imbedded, should be dissected out as an unbroken piece.

The following classification of the operations is made:—1. Complete ablation to the apex of the axilla, without removal of any pectoral muscle—two cases. 2. Complete ablation to the apex of the axilla with removal of the pectoralis major only—eleven cases. 3. Complete ablation to the apex of the axilla with removal of both pectoral muscles—twelve cases. 4. Complete ablation to the apex of the axilla with removal of one or both pectoral muscles, and invasion of the supra-clavicular region—eighteen cases. Of the two cases in Class 1, the first remained well for six years after the operation, when symptoms of intra-thoracic disease developed and proved fatal. In the second case local recurrence occurred 18 months later in the pectoralis major muscle, and after this had been removed the patient was well for five years, when cancer developed in the remaining breast. This was then operated upon but the patient subsequently died of carcinoma of the liver, ten years after the first operation on the breast. Of the 11 cases in Class 2 four remained well for periods of from eight to ten years, two remained well for six or more years and then got recurrence, three had recurrence within three years, one was well 12 months after the operation but subsequently was lost sight of, and one died of cerebral apoplexy, one year after the operation, without signs of recurrence. Of the five cases in which secondary growths developed in only two were these deposits in the region of the primary tumour. In these instances included in Classes 1 and 2, Dr. Pilcher remarks that the malignant growths were less far advanced than in the other classes. In Class 3 are included 12 cases and the results are as follows:—One lost sight of, one died five years after the operation of acute pneumonia, there being no sign of recurrence. One has remained well for three years. Three are alive three to five years after operation but have recurrence. Six died of recurrent growths one to five-and-a-half years after the primary operation. In five of these last-mentioned cases the recurrent growths occurred in the supra-clavicular region. On account of this fact Dr. Pilcher thinks it is reasonable for practical purposes to regard the supra-clavicular lymphatic tissues as diseased in all cases in which the glands at the apex of the axilla are markedly affected, and that the rational procedure for the surgeon to pursue in all such cases is to open up the supra-clavicular space and clear it out in the same manner as the axilla. Accordingly he has carried out this method in 18 cases which are included in Class 4. In ten of these enlarged glands could be recognised in the supra-clavicular region before the space was opened up. In the remaining eight no enlarged glands could be felt before the deep fascia was divided, but in all of them cancerous glands were found at the operation and dissected out. Only two of all these 18 patients have remained well, and these two have passed respectively six and four and a-half years since the operation in good health, a very different degree of success to that obtained in the earlier and less advanced cases in Classes 1 and 2. It will be seen that of the 13 less advanced cases included in Classes 1 and 2, 10 lived for over six years after the first operation and four of these were free from recurrence at periods of 8 to 10 years after the primary growth was removed. Of the remaining three, one died of apoplexy without signs of recurrence, one was lost sight of, and only one of the whole 13 died of recurrence within six years of the primary operation. Many of the more advanced cases had been under the observation of their medical attendant for months before being advised, or before they made up their own minds to have an operation done. These results show in a striking manner how disastrous it is to defer operation in cases of mammary tumour of doubtful nature until the malignancy of the growth is manifest or until the patient is driven to seek relief on account of the pain or some other distress caused by the extension of the disease. To quote Dr. Pilcher's own words: "It cannot be too strongly emphasised that practically every case of carcinoma of the breast, when it has reached that degree of development by which a palpable tumour is formed, is already in an advanced stage."

